# The structure and photoelectrochemical activity of Cr-doped PbS thin films grown by chemical bath deposition

**DOI:** 10.1039/c9ra11042a

**Published:** 2020-04-09

**Authors:** Ashour M. Ahmed, Mohamed Rabia, Mohamed Shaban

**Affiliations:** Nanophotonics and Applications (NPA) Lab, Physics Department, Faculty of Science, Beni-Suef University Salah Salem Street Beni-Suef 62514 Egypt ashour.elshemey@gmail.com ashour.mohamed@science.bsu.edu.eg; Polymer Research Laboratory, Chemistry Department, Faculty of Science, Beni-Suef University Beni-Suef 62514 Egypt

## Abstract

Nanocrystalline undoped and Cr-doped PbS thin films were prepared on glass substrates by a simple chemical bath deposition method. The X-ray diffraction analyses of the films showed their polycrystalline nature with cubic structure and preferential growth along the (111) orientation. Cr incorporation decreases the average PbS crystallite size from 59.97 to 37.59 nm, whereas the strain and dislocation density showed an increasing trend. The atomic ratio of doping for Cr is about 0.63, 1.75, and 4.70% according to energy-dispersive X-ray (EDX) spectroscopy. Morphological analysis showed that the average sizes of nanoclusters decreased from 73 to 41 nm as the Cr concentration increased. The optical band gap values are increased with increasing Cr doping. The photoelectrochemical (PEC) behaviors and the stability of the Cr doped PbS photoelectrodes were studied in 0.3 M Na_2_SO_3_ electrolyte solution. Also, the incident photon-to-current efficiency and applied bias photon-to-current efficiency are calculated and showed optimized values of 13.5% and 1.44% at 0.68 V and 390 nm. Moreover, the optimized electrode shows high chemical stability and a long lifetime. Finally, the effect of temperature on the PEC behaviors is evaluated and the different thermodynamic parameters are calculated.

## Abstract 2

Nanocrystalline undoped and Cr-doped PbS thin films were prepared on glass substrates by a simple chemical bath deposition method as photoelectrodes for solar water splitting.

## Graphical abstract

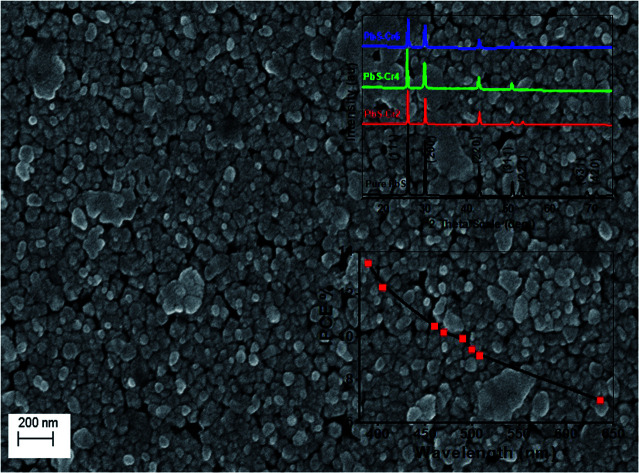

## 1. Introduction

Today, the energy crisis is one of the big problems facing humanity. Finding renewable and clean energy sources is urgently needed to satisfy increasing human demands.^1^ A hydrogen fuel cell is an excellent solution to meet the future global energy demand.^2^ In this fuel cell, the hydrogen can react with oxygen in the air to release energy and produce water as a by-product. The photoelectrochemical (PEC) method for H_2_ generation from water utilizing a semiconductor photoanode under sunlight is expected to make a significant contribution to face the energy crises in the future.^3^ The hydrogen production by this PEC technology is clean and doesn't produce any dangerous pollutants. The efficiency of PEC for hydrogen production depends on the performance of photoelectrodes such as its ability of light absorption and charge separation surface area for the electrochemical reactions.^4,5^ On the other hand, the lead sulfide (PbS) is an important p-type semiconductor material. It has a narrow direct band-gap, high dielectric constant, high absorption coefficient, large Bohr radius and very high carrier mobility.^6–8^ When the size of the PbS nanoparticles is below the Bohr radius, it leads to the quantum confinement effect of both electrons and holes. This confinement induces discrete electronic states in the valence band (VB) and conduction band (CB) of the nanoparticles compared to the continuous state of energy in bulk material.^9^ Therefore, the quantum confinement provided strong control of the bandgap value by changing the crystallite size according to the effective mass model.

PbS has the efficient light-absorbing capacity from the visible to near-infrared due to the bandgap of PbS can be tuned across visible to NIR region.^10,11^ Hence, PbS has good photocatalytic efficiency of hydrogen production.^12^ Also, the PbS nanoparticles can also show multiple exciton generations (MEG).^13,14^ In the MEG process, two electron–hole pairs (excitons) are produced by the absorption of one high-energy photon, bypassing hot-carrier cooling *via* phonon emission.^15^ This phenomenon was used to improve the photoconversion efficiency.^16,17^ Moreover, PbS possesses third-order nonlinear optical (3NLO) response 30 times that of GaAs and 1000 times that of CdS nanoparticles for particles of a similar size, which makes the PbS nanoparticles are suitable for optoelectronics applications.^6,18^

Many authors have presented a great interest in the study of the doped PbS thin films with various metal elements^19–21^ to tuning their optoelectrical properties from the viewpoints of practical applications. The impurity atoms provide sharp atomic levels for capturing exciton electrons and holes from the host lattice which help in tuning the properties of PbS. The nature and concentration of the dopant are responsible for the improved physical properties and efficiencies of PbS based semiconductor devices.

Gulen reported manufacturing Fe-doped PbS films on glass substrates by the SILAR method. The optical bandgap of the PbS films decreased from 1.66 to 1.25 eV as the Fe-doping concentration is increased from 1 to 9% Fe-doped PbS.^22^ Touati *et al.* have detected a blue shift of the bandgap due to the quantum confinement effect for (0–8%) Ag-doped PbS thin films.^23^ A systematic decrease in the crystallite size with increasing Hg concentration in the PbS thin films was reported by Palomino-Merino *et al.*^24^ Also, Portillo *et al.* showed that wide bandgap could be obtained for PbS nanocrystals doped *in situ* simultaneously with Er–Cd–Bi.^25^ In addition, the optical absorption of Mn, Cd and co-doped PbS nanocrystals have been investigated by Saravanan *et al.*^26^

Most of the previous work focused on enhancing the PEC properties by deposited PbS nanoparticles on different photoelectrodes such as ZnO, TiO_2_, and CuO–Fe_2_O_3_. Hsu *et al.* studied Al-doped ZnO nanorod thin film decorated with PbS nanoparticles as a photoelectrode for solar water splitting.^16^ The enhancement in the photocurrent density is due to the lowering of energy bandgap. Zhao *et al.* introduced a green synthesize of near-infrared PbS/CdS core/shell quantum dots on TiO_2_ mesoporous photoanode for photocatalytic hydrogen production.^27^ Shi *et al.* studied the multiple exciton generations using ZnO@PbS/graphene oxide photocatalyst for H_2_ production from water. The H_2_ evolution efficiency is remarkably improved by the synergistic effect of multiple exciton generations combined with the electron–hole separation of graphene oxide.^28^

Although the previous attempts, there are substantial needs for low cost, stable, and more efficient photoelectrochemical electrodes toward the industrial hydrogen production. However, less work was devoted to the photoelectrochemical properties based on PbS thin films.

On the other hand, chromium (Cr) is a typical transition metal element with a relatively abundant in the earth's crust. This element can be used in many applications such as dyes, paints, stainless steel alloys, a protective layer, blast furnaces, artificial rubies, catalysts for processing hydrocarbons, and recording magnetic tapes due to its physicochemical properties. The Cr can enhance the interaction with light because it possesses surface plasmon interactions.^29^ Also, chromium metal crystallizes as a body-centered cubic lattice with a lower energy of the high spin configuration. In addition, it has abundant electrons in the outer shell of its electronic configuration. Hence, it exhibits several oxidation states, ranging from −4 to +6.

In a previous literature report, the oxidation state of Cr ion in the Cr-doped PbS sample is +3 (ref. 30). The +3 state is a common oxidation state of Cr due to it is the most stable energetically according to crystal field theory (CFT).^31,32^

The Cr^3+^ has an ionic radius very small compared to that of the Pb^2+^ ion. The electronegativity of Cr (1.66) is lower than that of Pb (2.33) on the Pauling scale. Hence, incorporation of Cr^3+^ ions into the host PbS lattice permits more dopant ions to be accommodated in the Pb^2+^ sites thereby influence its physical properties. In many previous studies, the photoelectrochemical activity of photoelectrodes such as TiO_2_ and Fe_2_O_3_ show significant enhancement by doping with Cr due to an improvement in charge carrier properties.^33^ To the best of our knowledge, the photoelectrochemical studies on the Cr-doped PbS thin films have not been reported in the literature.

In the present work, Cr was chosen as a dopant for PbS thin films. Cr-doped PbS thin films with different concentrations of Cr were prepared by chemical bath deposition (CBD) method. The CBD is simple, low cost, good quality material and uniform coating at low temperature. The effect of Cr doping on the morphological, structural and optical properties of PbS thin films was investigated, and the results are presented. Also, the photoelectrochemical behaviors of the Cr-doped PbS films for the generation of H_2_ under visible irradiations are addressed.

## 2. Experimental details

### 2.1. Fabrication of PbS thin film

Commercial glass substrates were degreased in sulfuric acid (H_2_SO_4_) for 30 min and then immersed in isopropanol ultrasonically for 20 min. Finally, they have cleaned again ultrasonically with distilled water for 10 min and dried in air. The pure PbS and the Cr-doped PbS thin films were prepared by chemical bath deposition (CBD) in an alkaline medium.

For the deposition of PbS thin films, lead nitrate Pb(NO_3_)_2_ and thiourea CS(NH_2_)_2_ were used as the source of cationic Pb^2+^ and anionic S^2−^ precursor solutions, respectively. Sodium hydroxide NaOH was used as the complexing agent, which helps to control the reaction rate and prevent the formation of hydroxide precipitates. All the chemicals were analytically pure, and all aqueous solutions were prepared using DI water.

In a typical reaction, the bath solution was prepared by the sequential addition of 90 ml (10 mM) of lead nitrate and 90 ml (57 mM) of thiourea mixed with 90 ml (146 mM) of sodium hydroxide. These molar ratios are optimized after many experimental trials using varied ratios to obtain homogenous films with high uniformity and adhesion. It is worth to mention that not all thiourea are simultaneously decomposed to give S atom, so the molar ratio of thiourea is higher than the molar ratio of the lead nitrate. The solutions mixed in this order to obtain a final dark solution, a change may provide a different product at the end. The solution was stirred for 2 minutes to get a homogeneous solution. The final pH of the mixture was measured to be 12.8. The cleaned glass was vertically immersed in bath solution for 30 min at room temperature without stirring. In the beginning, the deposition bath was colorless, and its color has changed from brown to dark grey with time deposition.

The following eqn (1)–(5) give the reaction process for forming PbS film.^34^

1 Pb(NO_3_)_3_ + 2NaOH → Pb(OH)_2_ + 2NaNO_3_

2 Pb(OH)_2_ + 4NaOH → Na_4_Pb(OH)_6_

3 Na_4_Pb(OH)_6_ → 4Na + HPbO_2_^−^ + 3OH^−^ + H_2_O

4 SC(NH_2_)_2_ + OH^−^ → CH_2_N_2_ + H_2_O + SH^−^

5 HPbO_2_^−^ + SH^−^ → PbS + 2OH^−^

### 2.2. Fabrication of Cr-doped PbS thin films

For the fabrication of PbS thin films doped with Cr impurities, chromium trioxide (CrO_3_) acted as a source of Cr ions. Different Cr-doping levels were obtained by changing the volume of the Cr reagent solution into the PbS growing solution. PbS films with three different levels of doping Cr were obtained by the addition of 2, 4 and 6 ml of 1 mM CrO_3_ into sodium hydroxide in the above bath. Adequate molarity of 1 mM CrO_3_ of the doping solution was determined after several trials. The samples were labeled as PbS-Cr*x* (*x* = 0, 2, 4 and 6) for doped films, where the numbers 0–6 corresponds to the volume of CrO_3_ added to the PbS bath.

After deposition, the films were cleaned by rinsing with distilled water and then dried in air. Mirror-like thin film surface was obtained after removal of the deposited film from one side of the glass slide using cotton with dilute H_2_SO_4_. Finally, the films were annealed in air at 250 °C in a furnace for 2 h to improve the crystallinity of the films. The annealed films appear to be smooth, homogeneous and well adherent to the glass substrates.

### 2.3. Characterization

The surface morphologies of Cr-doped PbS thin films were characterized using field emission scanning electron microscope (FE-SEM, ZEISS SUPRA 55 VP, and ZEISS LEO, Gemini Column). The crystallization behavior was analyzed through an X-ray diffractometer (PANalytical X'Pert Pro, Holland) using CuKα radiation with operating voltage at 40 kV and 40 mA in scan range from 5° to 80° with a scan rate of 0.5° sec^−1^ and step size 0.04°. The energy dispersive X-ray spectrometer (JEOL JED-2300 SEM) was used to characterize the chemical compositional of Cr-doped PbS thin films. Optical spectra in the range from 300 to 2000 nm were studied with increment 1 nm using double beam PerkinElmer Lambda spectrophotometer at room temperature.

### 2.4. Photocatalytic activity test for hydrogen generation

Pure and Cr-doped PbS was used as a working electrode for photocatalytic hydrogen electro-generation experiments. Platinum (Pt) sheet was used as a counter electrode. 150 ml of 0.3 M Na_2_SO_3_ was used as the source electrolyte. The photoelectrochemical current–voltage and current–time curves were recorded with a Keithley Electrometer using LabTracer software (2400 SourceMeter, A Tektronix Company). The cell was illuminated with 500 watt mercury-xenon light source (Newport, 66926-500HX-R07) provided with a series of linear optical filters.

## 3. Results and discussion

### 3.1. XRD study and EDX analysis

**Fig. 1:**
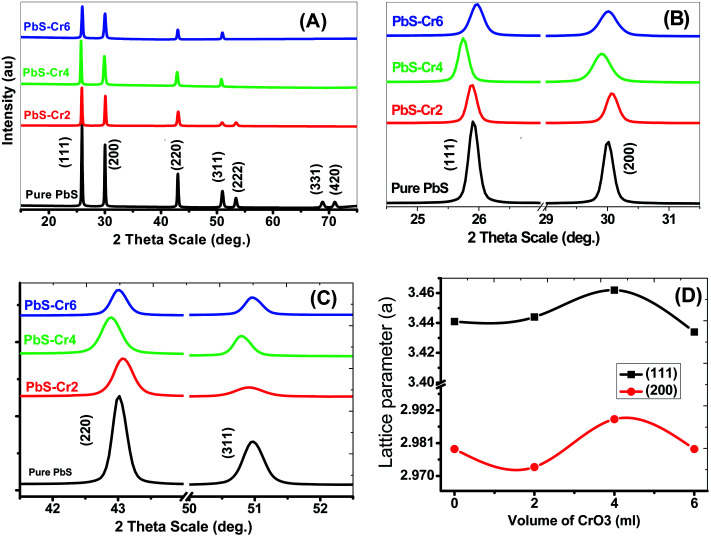
(A–C) XRD spectra for nanostructure pure and doped PbS thin films with different Cr content. (D) The lattice parameter for (111) and (200) planes.

The crystal structure of the pure and Cr-doped PbS thin films deposited on glass by the CBD was studied by XRD to identify the phases present. Fig. 1 shows the XRD patterns of pure and doped PbS films. According to standard cards # 04-002-0034 and 00-005-0592, all the observed diffraction peaks have single-phase agreed with the standard diffraction patterns of face-centered cubic PbS (space group *Fm*3̄*m*). Also, all films exhibit polycrystalline structures and show a strong (111) diffraction peak. The high intensity of the narrow peaks indicates the synthesis of nanosized films with good crystallinity.

There exist three variants to include Cr atoms in PbS nanoparticles. First, Cr can substantially replace Pb. Second, Cr atoms can occupy interstitial positions. Third, Cr atoms can take their places between the layers of PbS (intercalation) or take place on the internal and external surfaces of nanoparticles.

No additional XRD peaks were observed for Cr metal or Cr oxide due to the low amount of Cr-loading, which is below the detection limit of XRD.

The Cr doping did not significantly change the phase structure of the PbS crystal but the neighboring atoms of the Cr atom remain under tensile stress because the substantial Cr^3+^ ions (*R*_Cr^3+^_ = 0.615 Å) are smaller in size compared to the original Pb^2+^ ions (*R*_Pb^2+^_ = 1.19 Å). Similar results were obtained by Kayani *et al.* for ZnO thin films doped with Cr and prepared by sol–gel dip-coating method.^35^

Moreover, the structural XRD parameters for the highest four peaks; (111), (200), (220) and (311); are calculated and mentioned in Table 1. The crystallite size (*D*), lattice parameter (*a*), texture coefficient (TC), interplanar distance (*d*), strain (*ε*), and dislocation constant (*δ*) are calculated by using the following equations;^36,37^

6 *D* = 0.94*λ*/*β* cos *θ*

7
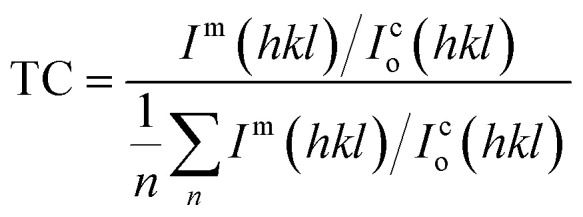

8 *d* = *λ*/2 sin *θ*

9
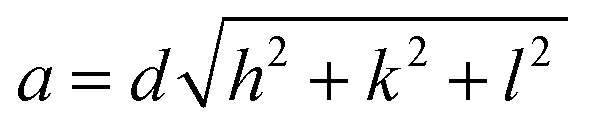

10 *ε* = 0.25*β* cot *θ*

11 *δ* = *D*^−2^

where *β* is full width at half maximum in radians, *θ* is the Bragg's angle, *λ* is the X-ray wavelength (CuKα = 0.154 nm). The *I*_m_(*hkl*) and *I*^c^_o_(*hkl*) are, respectively, the measured intensity and the standard intensity of (*hkl*) plane based on the standard data file (# 00-005-0592), and *n* is the number of diffraction peaks considered for analysis.

**Table 1:** The structural parameters of pure and Cr-doped PbS films from XRD data; crystallite size (*D*), lattice parameter (*a*), texture coefficient (TC), interplanar distance (*d*), strain (*ε*), and dislocation constant (*δ*)

Film	*hkl*	TC	*D* (nm)	*d* (Å)	*a* (Å)	*ε* × 10^−2^	*δ* ×10^−4^ (nm^−2^)
PbS (pure)	(111)	2.6	63.873	3.441	5.960	26.937	2.4510
	(200)	2.0	64.390	2.979	5.959	23.137	2.4118
	(220)	1.1	51.672	2.104	5.951	20.359	3.7452
	(311)	0.51	32.172	1.792	5.946	27.863	9.6610
PbS-Cr2	(111)	2.17	63.870	3.444	5.966	26.964	2.4512
	(200)	1.67	49.793	2.973	5.947	29.859	4.0331
	(220)	0.77	31.212	2.101	5.943	33.663	10.264
	(311)	0.18	21.189	1.795	5.953	42.356	22.271
PbS-Cr4	(111)	1.93	63.855	3.462	5.995	27.114	2.4524
	(200)	1.14	30.055	2.989	5.979	49.739	11.070
	(220)	0.57	31.192	2.110	5.967	33.822	10.277
	(311)	0.34	43.606	1.798	5.964	20.624	5.2590
PbS-Cr6	(111)	1.70	40.422	3.434	5.947	42.476	6.1199
	(200)	1.31	30.062	2.979	5.959	49.556	11.064
	(220)	0.56	42.312	2.104	5.952	24.868	5.5855
	(311)	0.40	43.637	1.792	5.946	20.542	5.2515

From the calculation data, the average crystal size (*D*) for the planes (111), (200), (220) and (311) of the pure PbS is found to be 53.026 nm and decreases to 39.108 nm with increasing Cr doping ratio to 6.0 ml due to the diffraction peak broadening of PbS thin films and induced strain. The peak broadening can result from an increase in heterogeneity of the films due to the occupation of Cr^3+^ into the host lattice.^24^ The crystallite and grain sizes play an important role in the optical properties of the films. The peak intensity of XRD depends strongly on the Cr percentage, which decreases with an increase in Cr concentration. For the main peak (111), the diffraction intensity peak decreased from 159.82 to 56.31 acts as a concentration increase from 0 to 6 ml.

The texture coefficient (TC) was evaluated to quantify the preferred crystallographic orientations of the samples. It was observed from Table 1 that the plane (111) has a high texture coefficient, indicating that the nanocrystals have preferential coordination towards the (111). In many previous works, the (200) preferential growth was observed for PbS thin films.^34,38^ The variation of preferred orientation can be explained from the thermodynamic point of view. Xigui *et al.* have proposed that the reason for this variation is the change of the total system free energy during the film growth which is affected by the surface, interface and strain energy contributions.^39^ Moreover, the degree of these three energies contributions may be different under certain preparation conditions, which consequently leads to different preferred orientations.^40^

For all planes, increasing Cr ions content is associated with a decrease of the TC, as shown in Table 1, as a result of a decline in the crystal quality. The values of TC for (111) plane are decreased from 2.6 to 1.70 as the Cr volume content increased from 0 to 6 ml. The decrease of TC values reveals to the reduction of the film crystallinity. Hence, the crystal quality deteriorates with increasing Cr concentration. Previously, Haazen observed that the crystal quality of the insulator Bi_2_Se_3_ weakens with increasing Cr concentration.^41^

In the case of pure PbS film, main peaks are assigned to (111), (200), (220), (311) planes at 2*θ* = 25.89°, 29.98°, 42.98°, and 50.93° respectively. Also, the presence of the other peaks such as (222), (331), and (420) was identified with lower intensities. The number of diffraction peaks is decreased from 7 to 4 peaks with increasing of the Cr percent from 0 to 6 ml in the PbS films. So, the increase of Cr concentration in the precursor suppressed the crystal growth of the PbS in the (222), (331) and (420) directions. The Cr ions may have terminal OH^−^ ligands in solution. Therefore, increased Cr concentration affects the concentration of OH^−^ ions that are consumed for thiourea (TU) decomposition and subsequent release of free S^2−^ ions.

The peak position of planes (111) and (311) are moved towards lower angles with increasing of the Cr percent in the films from 0 to 4 ml, but after that, the position moved towards higher angles. This shift can be attributed to doping-induced structural lattice deformation and disorder in the PbS crystal. Lattice constant changes are closely correlated to the shifts of the 2theta positions of XRD peaks. As a result of the increase of the lattice, the diffraction peaks are shifted towards lower Bragg angles. The changes of the lattice constant can be explained through three doping-sensitive factors: size effect, electronic deformation of the consistent conduction band minimum, and (iii) doping-induced variations of the lattice vibration anharmonicity. The distribution of Cr^3+^ in PbS lattice changes with increasing Cr concentration. Cr-doped PbS films with low Cr concentration could reach equilibrium. Accordingly, the low Cr concentration to a certain degree disorder the crystal lattice relative to the pure PbS lattice. Hence, the lattice constant increased and the diffraction angle decreased. By increasing the Cr% the distribution of the Cr^3+^ improved. Since the radius of the substantial Cr^3+^ ion is smaller than the radius of the original Pb^2+^ ion. Then, a larger Cr doping (>4%) should decrease the lattice constant (Table 1) due to the tensile stress on the neighboring atoms and structural relaxation. As a result, the diffraction angle is increased.

The interplanar distance (*d*) and lattice parameter (*a*) are increasing with increasing the Cr percent in the films from 0 to 4 ml but after that it with Cr percent. These due to the angle of diffraction *θ* is inversely related to the interplanar spacing *d* (sin *θ* is proportional to 1/*d*) according to Bragg's law.

The increase in the lattice parameter is not expected because the ionic radius of Cr^3+^ (0.63 Å) is smaller than that of Pb^2+^ ions (1.01 Å). In Cu-doped Bi_2_Se_3_, the copper (with an ionic radius of 0.71 Å) is substituting the bismuth (with an ionic radius of 1.17 Å) in the lattice and located between quintuple layers inside the van der Waals gap of the crystal structure.^41^ A similar mechanism is introduced to explain the increase of the lattice parameters (*a*) in the Cr-doped films.

The obtained lattice parameter ‘*a*’ values in Table 1 are found to be slightly higher than the standard value (5.9362 Å) due to the presence of intrinsic defects and loss of stoichiometry of the synthesized PbS-Cr films.

The lattice parameter ‘*a*’ has a nonlinear relationship with the content of Cr^3+^ for (111) and (200) planes, which not follow Vegard's law (Fig. 1(D)). This result demonstrates that the Cr ions are incorporated nonhomogeneous into the host PbS matrix due to the difference in the ionic radius and chemical oxidation states.

Increasing in lattice parameter with an increase in Cr^3+^ percentage produces expansion in the PbS unit cell. Therefore, the high strain is induced in crystal, and hence, crystalline qualities are deteriorated. The origin of strain is related to lattice misfit which in turn depends upon the growing conditions of the films.^42^ The strain increased from 0.2457 for pure PbS to 0.3436 for PbS-Cr6.

Dislocations are imperfections in a crystal that associated with misregister of the lattice in one part concerning another part of the crystal. The dislocation density (*δ*) defined as the length of dislocation lines per unit volume of the crystal. The value of *δ* for Cr-doping PbS films is higher than that of pure PbS film, which supports the increment in the number of defects in the doped PbS films. The increase in dislocation density suggests that films become less crystalline.^43^ Therefore, the presence of dislocations strongly influences many properties of the prepared materials.

**Fig. 2:**
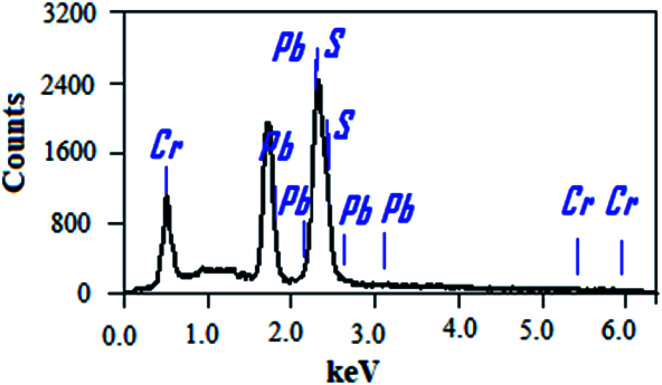
EDX spectrum of PbS-6Cr film.

The chemical compositions of the PbS and PbS-Cr films were studied by energy-dispersive X-ray (EDX) spectrometry. Fig. 2 shows the EDX spectrum of the PbS-6Cr film. The quantitative analysis of the PbS and PbS-Cr films are shown in Table 2. The EDX patterns indicated the presence of Pb, S and Cr signals as the components of the synthetic thin films as seen in Fig. 2 and Table 2. No impurities are detected in the EDX patterns, which indicate the films are well-crystallized and they are in good agreement with the XRD results. The ratio of Cr in the PbS-Cr films increased from 0 to 4.70% as the volume of CrO_3_ added to the PbS bath increasing from 0 to 6 ml. The atomic ratio of Pb : S : Cr is 42.92 : 52.39 : 4.70 for the PbS-Cr6 film.

**Table 2:** The EDX analysis of PbS-Cr film at different levels of Cr doping varied from 0 to 6 ml

Film	Element		
	Pb atomic%	S atomic%	Cr atomic%
PbS	44.43	55.57	0
PbS-Cr2	46.11	53.26	0.63
PbS-Cr4	43.01	55.24	1.75
PbS-Cr6	42.92	52.39	4.70

### 3.2. SEM morphology

**Fig. 3:**
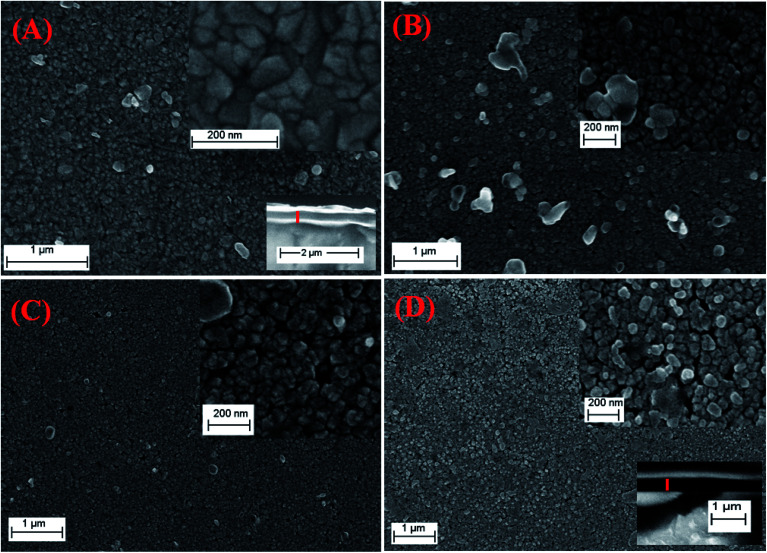
SEM micrographs for (A) pure PbS, (B) PbS-Cr2, (C) PbS-Cr4 and (D) PbS-Cr6 thin films. The lower insets of (A) and (D) are cross-sectional SEM images.

The morphologies of pure and Cr-doped nanostructure PbS thin films were studied using field emission-scanning electron microscopy (FE-SEM) as seen in Fig. 3. Pure PbS nano-stones are coalescent and intergrown with each other to form a continuous layer of multi-shape nano-stones, Fig. 3(A). These nano-stones have a random distribution with irregular shapes and sizes leading to rough PbS surface.

The doping with Cr was observed to modify the surface morphologies and grain sizes, Fig. 3(B–D). It is clear that the surfaces of Cr-doped PbS films are formed from nucleation and coalescence of nanoparticles close to spherical granules with different diameters. The Cr-doped PbS nanoparticles are self-assembled to generate different voids with varied diameters.

As the Cr concentration increased, the average size of nanoparticles and the density of nanoparticles are decreased. The average size of nanoparticles decreases from 73 to 41 nm as the Cr volume content increased from 2 to 6 ml. This due to the Cr^3+^ ions consumes the OH^−^ ions and hence reduces the growth rate. Therefore, the sizes of nanoparticles and the cluster of nanoparticles tend to be smaller and homogenize as the doping level increased. This means that the surface area of Cr-doped PbS films increases with increasing the doping level. The thicknesses of the films are increased from 320 nm for pure PbS to 450 nm for PbS-Cr6 as seen in the inset cross-sectional images of Fig. 3(A and D). Similar results were reported for Cr-doped CdO and Cr-doped ZnO thin films.^44,45^

### 3.3. Optical properties

**Fig. 4:**
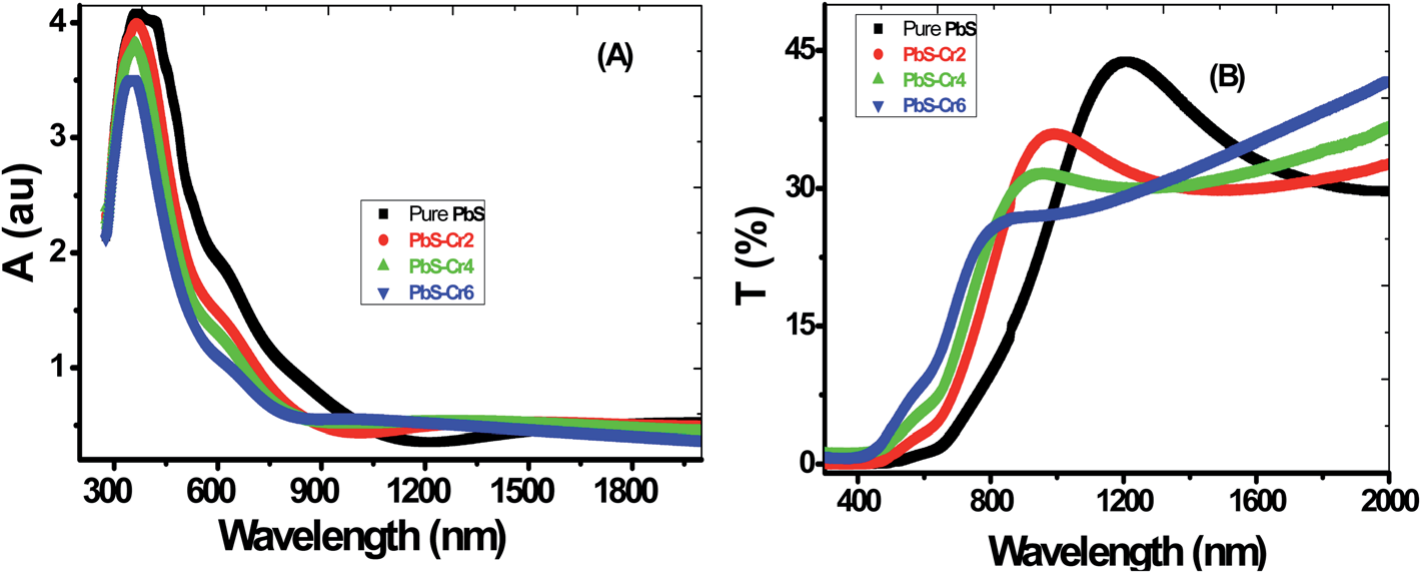
Optical (A) absorbance (B) transmittance of pure and Cr-doped nanostructured PbS thin films.

The absorbance (*A*) and transmittance (*T*%) spectra from 300 nm to 2000 nm of all the films are shown in Fig. 4(A and B). During the scanning process, a blank glass slide was placed in one of the beam direction, and another glass with the deposited PbS film was in the other beam's direction. Thus, the absorption and transmission spectra displayed by the spectrophotometer were as a result of the films deposited on the glass slides.

All samples have two absorption bands; a sharp peak at UV region and a small broad one at the NIR region. These peaks attributed to band-gap absorption of PbS and structural defect absorption, respectively.^46^ The absorbance decreases sharply with wavelengths ranged from 300 nm up to 900 nm. The sharper peaks in the absorption spectra for all PbS films indicate a narrow size distribution of the nanoparticles. These spectra show an abrupt change in the doped samples compared to pure PbS. As seen in Fig. 4(A), the absorbance is decreased by increasing the Cr doping concentration. This is accompanied by a color change of PbS films from dark grey to red-grey with increasing Cr ratio. Jiabin Huo *et al.* observed similar behavior for Cr-doped PbS nanofilms.^30^ The absorption edge gradually shifted from longer wavelengths (near IR region) to shorter wavelengths (visible region) in the doped films signifying an increase in their bandgap values. These shifts are due to the decrease in the crystallite size as a result of the confinement effect. A clear absorption edge shown by all the grown thin films confirms the crystalline nature of the material.

From Fig. 4(B), the general behavior of transmittance spectra can be divided into three regions. At low wavelength in the UV region, all the PbS thin film having a nearly zero transmittance, which is due to strong absorbance in this region. In the medial part of spectra (550–1000 nm), the transmittance of the films varies linearly with wavelength. In the NIR region, a slight decrease in transmittance consequently for high wavelength electronic transitions are existing in this wavelength range. It can be seen that PbS film has a transparency of about 30% in the near IR region of the spectrum. Touati *et al.* obtained the same behavior for PbS thin doped with Cu metal.^47^

The film's transparency of pure PbS below 900 nm increases with increasing Cr doping ratio. This behavior can be attributed to the grain growth seen in SEM results, giving rise to less scattering effects from the grain boundaries. Another reason for the increased transmittance that observed for the doped films in the UV/visible light region might be due to change in free carriers. Each incorporated Cr^3+^ ion brings one free electron in the PbS lattice when substituting the Pb^2+^ ion thereby increasing the carrier concentration. *I.e.*, Cr atoms substitute Pb atoms and introduce additional free electrons in the parent PbS lattice. Although PbS is a p-type material, however, the Cr-doped PbS nanofilm acts as an n-type semiconducting material due to the incorporation of the Cr donors.

Also, the transmittance spectrum is blue-shifted with an increase in Cr doping concentration.

Based on recorded absorbance (*A*) and film thickness (*d*), the absorption coefficient (*α*) was calculated using the relation:

12 *α* = 2.303*A*/*d*

The type of transition associated with the band structure of PbS can be identified using the following Tauc equation

13 (*αhν*)^2^ = *K*(*hν* − *E*_g_)

where: *K* is an energy-independent constant, *h* is Planck's constant, *ν* is the photon frequency, *E*_g_ is the optical band gap between the bottom of the conduction band and top of the valence band, and *α* is the absorption coefficient.

**Fig. 5:**
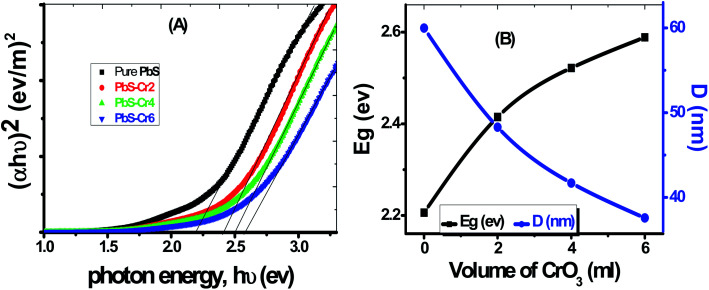
(A) Plots of (*αhν*)^2^*versus hν* to determine *E*_g_ of pure and Cr-doped PbS films, (B) dependence of *E*_g_ and *D* on the level of Cr doping.

The value of the direct bandgap energy has been calculated from the straight-line portion of the (*αhν*)^2^*versus hν* curve that intersects the *hν* axis, as shown in Fig. 5(A). The plot of *E*_g_*vs.* Cr doping concentration for all samples is displayed in Fig. 5(B). The *E*_g_ value of the pure PbS film was found to be equal to 2.196 eV, which matches with the value reported by Barote *et al.* for PbS thin film deposited by CBD method.^49^ The PbS-Cr2 film has a bandgap value of 2.422 eV. The *E*_g_ value is found to be blue shifted to 2.589 eV for the PbS-Cr6 film, which may be due to the rise in the free carrier concentration. The increase in *E*_g_ value with carrier concentration is ascribed to the Moss–Burstein effect, whereas the dopant element increases electrons density in the conduction band thereby lifting the Fermi level.^48^

According to the quantum confinement effect, the bandgap widening takes place when the particle size is decreased, as shown in Fig. 5(B). Hence the increasing *E*_g_ can be correlated with the decrease in average crystallite size of the main three peaks calculated from XRD data as shown in Table 1. The reduced grain size leads to a reduction of the scattering, and consequently, the optical band increased. In many previous works, the *E*_g_ was found to be varying inversely with the crystallite size.^50,51^ The value of *E*_g_ for these sets of films demonstrates that they are good semiconductor candidates for absorbing visible light in optoelectronic and photovoltaic applications.

### 3.4. Photoelectrochemical H_2_ generation

**Fig. 6:**
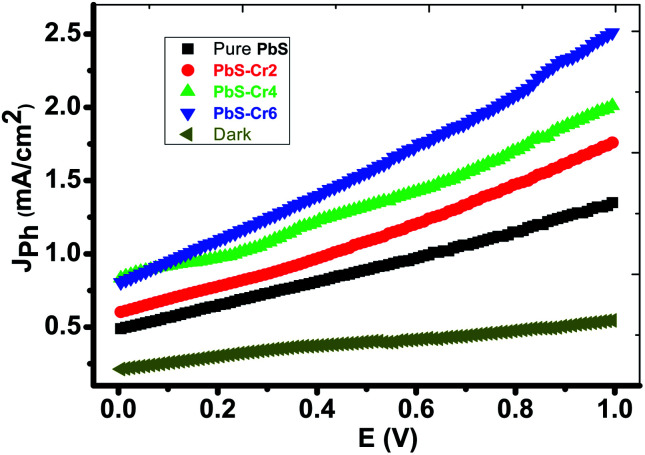
Cr-doped PbS electrodes photocurrent density–voltage curves supported on glass under the illumination of 500 W mercury-xenon Lamp without the optical filter.

Fig. 6 shows the photoelectrochemical (PEC) behavior of the Cr-doped PbS electrodes was measured under the illumination of a 500 W mercury-xenon lamp without an optical filter. A prepared 1 cm^2^ electrode is employed as the photoanode and a 1 cm^2^ Pt-electrode is used as the counter electrode. A 150 ml of 0.3 M Na_2_SO_3_ aqueous solution is used as a redox electrolyte for solar water splitting. The linear sweep voltammetry performed in the range of 0 to 1.0 V with a 1 mV s^−1^ sweep rate at room temperature.

From Fig. 6, it can see that the amount of Cr concentration plays a vital role in affecting their PEC properties. The current density is increased from 1.33 to 2.5 mA cm^−2^ at 1 V as the volume of CrO_3_ risen from 0 to 6 ml under light illumination. Therefore, PbS-Cr6 is the optimized PEC electrode, which produced the maximum photocurrent and showed the highest PEC properties. Also, it is observed that the photocurrent density increases with applied voltage. The increase in current can be attributed to the small contact height and an increase in tunneling mechanism.^52^

Although the increasing quantity of Cr impurities in PbS films decreases the light absorption, the efficient charge separation of the electron–hole pairs and suppressed recombination of photogenerated charge carriers of small PbS nanoparticles leading to the rise of photocurrent.^53^

Also, this increase in *J*_ph_ can be understood regarding the decrease in the size of the PbS-Cr nanoparticles (Fig. 2) as Cr doping increases. The reduction in the size of the PbS nanoparticles PbS-Cr6 sample will increase the conduction band edge due to a rise in the bandgap of the PbS nanoparticles which would lead to an increase in the driving force for the transfer of photogenerated electrons at the counter electrode.^54^

The increase of Cr contents induces one free electron into the PbS lattice. So, the carrier concentration increases simultaneously which enhances the photocurrent during water splitting. Also, the conductivity of PbS thin films increases remarkably on doping with Cr.^55^ Increasing the carrier concentration in the semiconductor reduces the space charge layer width, and to some extent, improves the tunneling probability of electrons across the potential barrier.^56^ Also, the enhanced charge carrier density can extend the lifetime of charge carriers, giving rise to a reduction in the recombination of the electron–hole pairs.^57^ In addition, the large surface area of the PbS-Cr6 electrode produces electron–hole pairs of high density, which will motivate the splitting of H_2_O molecules under the effect of light to carry out the hydrogen generation reaction.

**Fig. 7:**
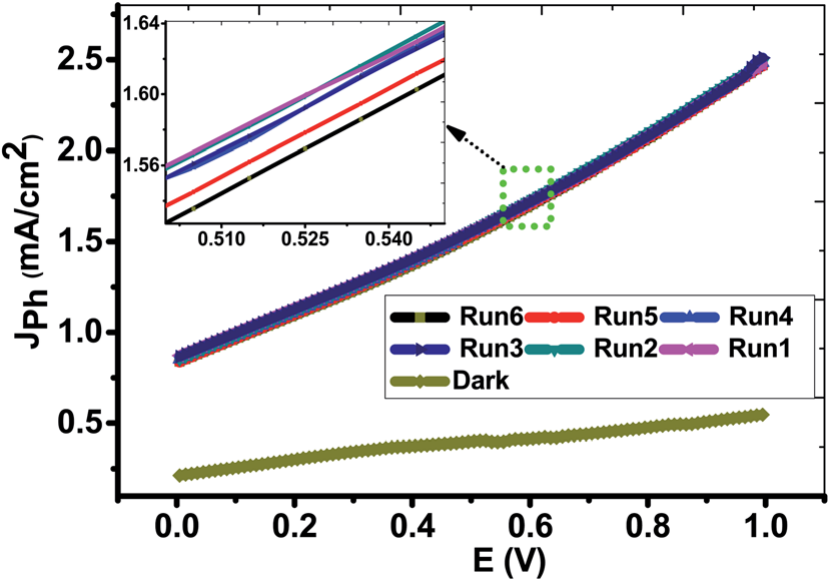
Reproducible studies of photocurrent density–voltage curves of the PbS-Cr6 electrode in dark and under white light illumination without the optical filter.

Form the experimental viewpoint; the reproducibility study is significant for approving the obtained data. Fig. 7 illustrated the *J*_ph_–*E* behaviors of the PbS-Cr6 electrode was measured in the dark and under the illumination of 500 W mercury-xenon lamp without an optical filter for many repeats. From this figure, the *J*_ph_ values for the PbS-Cr6 electrode are measured six times with relative standard deviation (RSD) of 0.63% and a mean value of 2.48 mA cm^−2^. Also, the photoelectrode shows a quite low dark current and negligible concerning their respective photocurrent, indicating no fierce electrocatalytic water splitting occurs.^53^

**Fig. 8:**
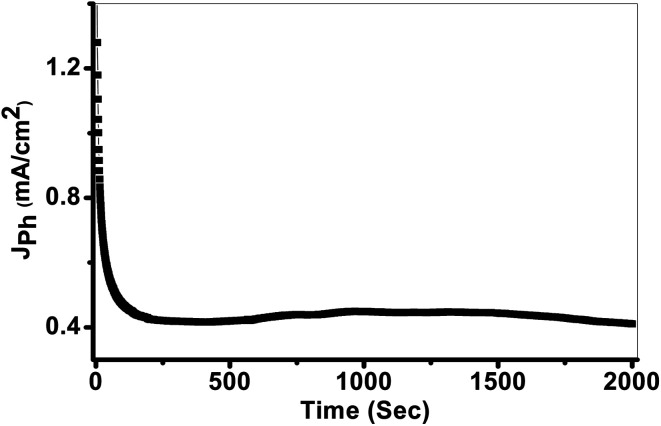
Current–time characteristic under white light illumination.

The photostability of the PbS-Cr6 electrode is investigated for an elongated time and shown in Fig. 8. During these measurements, a small bias voltage of 0.85 V is introduced between the electrodes to overcome any external losses of the measuring system. From Fig. 8, the *J*_ph_ values are decreased in the first period due to the minimal corrosion that occurs as a result of the first reaction of the electrode with the electrolyte. Also, the high surface density of states maybe results in a significant pinning of the Fermi level that can facilitate the participation of these defect states in the surface oxidation process, leading to small degradation of the PbS-Cr6 electrode. Above 200 s, the *J*_ph_ values remain constant nearly at 0.42 mA cm^−2^ due to increasing the accumulation of the ionic charges. Doping PbS film with Cr impurities leads to an increase of surface area and a decrease in the electron/hole recombination rate. Therefore, the significant increase in the photostability of PbS-Cr6 photoelectrode allows the reusability of the PbS-Cr6 for several runs. This result is suggesting that the photoanode has high chemical stability and a long lifetime to work in the H_2_ production cell.

**Fig. 9:**
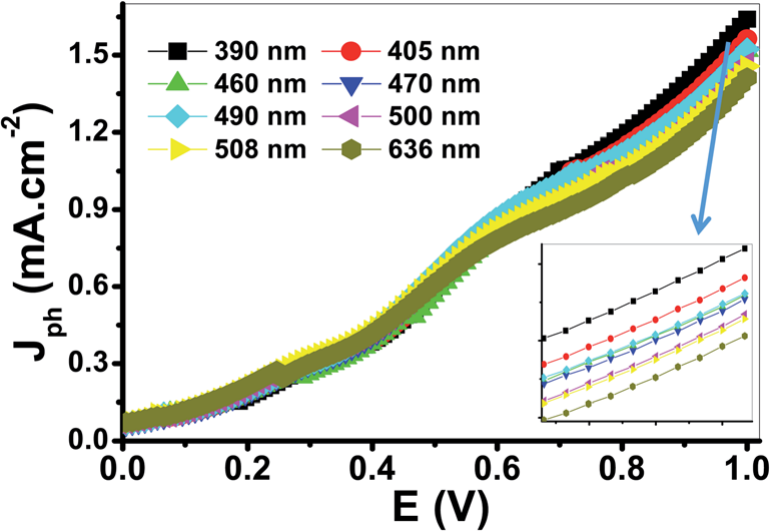
Photocurrent-voltage curves of the PbS-Cr6 electrode under the illumination of monochromatic light.

The variation of *J*_ph_ with the applied potential under the illumination of monochromatic light for the electrode is shown in Fig. 9. The average intensity of the incident monochromatic light was 50 mW cm^−2^. The *J*_ph_ values decrease with increasing the optical wavelength from 390 to 636 nm. The photoelectrochemical *J*_ph_ for water splitting has maximum and minimum values of 1.63 and 1.41, respectively. As the optical wavelength of the incident light decreased, the required potential for the photoelectrochemical generation decreases due to the photon energy increase.

The PEC performance of the PbS-Cr6 electrode is evaluated by measuring the incident photon-to-current conversion efficiency (IPCE) and photon-to-current efficiency (ABPE) under monochromatic illumination conditions. The IPCE analytical measurements give meaningful insight into the contribution of the PbS-Cr6 film in the conversion efficiency of the incident photons into charge carriers. A higher value of IPCE signifies improved transportation of photoexcited charge carriers. The IPCE is determined at an applied potential of 1 V from eqn (14).^58^14
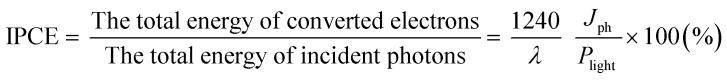
where *J*_ph_ is the photocurrent density at that particular wavelength of incident light (mA cm^−2^); *λ* is the wavelength of the illuminating monochromatic photon (nm) and *P*_in_ is the illuminating light power density (mW cm^−2^).

Based on the optical behavior of the PbS-Cr6 film, the prepared electrode has an optimum IPCE% value of 13.5% at 390 nm, then this value decreases with increasing the wavelength to reach 7% at 636 nm as shown in Fig. 9(A).

The ABPE represents the development of the photoelectrode performance as a function of the applied potential. It measures the photoconversion efficiency of light energy to chemical energy. The ABPE efficiency values for the designed photoelectrodes are calculated by using eqn (15):^59^15
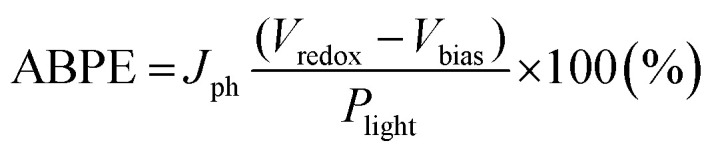
where *V*_redox_ is the redox potential for H_2_O splitting (1.23 V) *V*_bias_ refers to the actual applied potential difference between the working and the counter electrode; *P*_light_ is the light intensity (100 mW cm^−2^) and *J*_ph_ is the generated photocurrent density. For Fig. 9(B), as the applied potential increases, the ABPE% attains its maximum value at 0.68 V and 390 nm, but it decreases again when the applied potential approaches to the thermodynamic H_2_O potential (1.23 V). As the wavelengths of the incident photons increase from 390 to 636 nm, the ABPE efficiency values decrease from 1.44 to 1.27% and their position shift from 0.68 to 0.59 V. Finally, the obtained values of IPCE, ABPE, and *J*_ph_ of the present study are summarized with the previously reported values of many photoelectrodes as shown in Table 3. From this table, the reported values in this study for IPCE and ABPE are higher than those previously obtained for the displayed photoelectrodes.

**Table 3:** Comparison of IPCE and ABPE values of the present work with previously reported values for many photoelectrodes

Photoelectrode	Performance
Cr-doped PbS	*J*_ph_ = 1.5 mA cm^−2^, IPCE = 13.5%, ABPE = 1.44% at 390 nm (present study)
Trimethylphenylporphyrin	*J*_ph_ = 0.1 mA cm^−2^, IPCE = 0.028% (ref. 60)
ITO/PMPDI/CoO_*x*_	*J*_ph_ = 0.15 mA cm^−2^, IPCE = 1% at 1.6 V (ref. 61)
CuNb_3_O_8_	IPCE = 6–7% at *λ* > 420 nm (ref. 62)
Ag-doped CaFe_2_O_4_	IPCE = 3.8% (ref. 63)
Si-doped InGaN-based nanowire	*J*_ph_ = 1.42 mA cm^−2^, ABPE = 0.9% at 1.5 V (ref. 64)
Au/TiO_2_ nanotube	IPCE = 8% (ref. 65)
TiO_2_ nanotube	*J*_ph_ = 1.5 mA cm^−2^, ABPE = 0.84% at 100 mW cm^−2^ (ref. 66)
InGaN/GaN nanowire	*J*_ph_ = 1.8 mA cm^−2^, IPCE = 9% at 1 V (ref. 67)

**Fig. 10:**
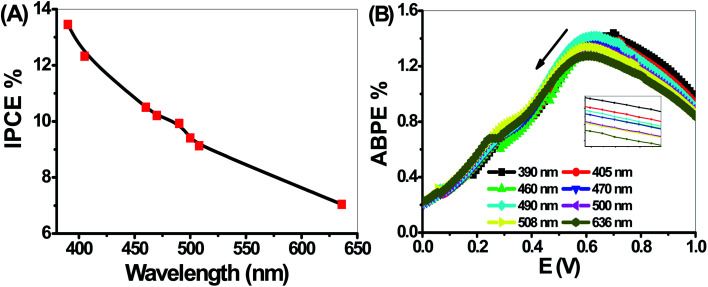
(A) IPCE% and (B) ABPE% as a function of wavelength for the PbS-Cr6 electrode.

**Fig. 11:**
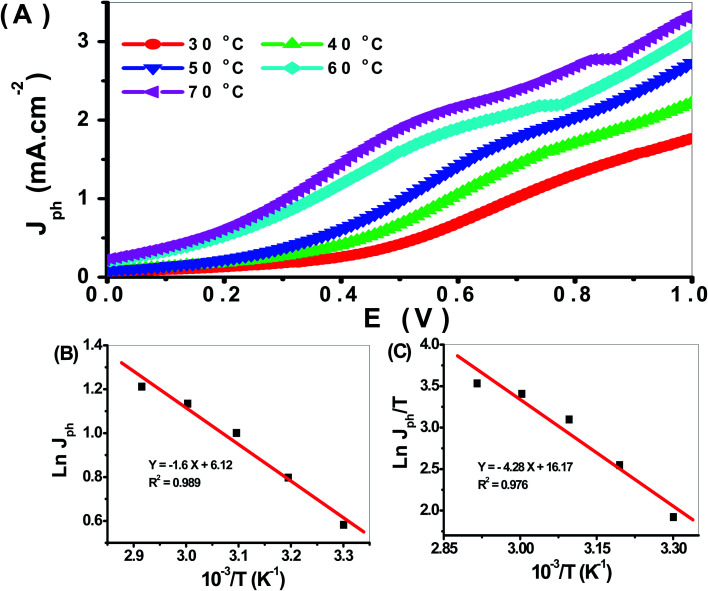
(A) The effect of temperature on H_2_ generation process, (B) plot of the *J*_ph_ against reciprocal of temperature, and (C) plot of *J*_ph_/*T versus* reciprocal of reciprocal temperature.

The effect of temperature from 30 to 70 °C on the PbS-Cr6 electrode for H_2_ generation is shown in Fig. 10(A). From this figure, the *J*_ph_ values increase with increasing temperature due to the increased mobility of ions. So the increase in temperature increases the H_2_ generation process. So the rate of H_2_ generation under different temperatures is related to the *J*_ph_ values. The relation between reciprocal of temperature and *J*_ph_ values (rate of reaction) is represented in Fig. 10(B). From this relation, the *E*_a_ can be calculated using the Arrhenius equation. From the slope value, the *E*_a_ value is 24.32 kJ mol^−1^. This value is small, so the prepared electrode is very efficient for the H_2_ generation reaction. In addition, the enthalpy ΔH* and entropy ΔS* for the H_2_ generation reaction can be calculated using the Eyring equation and Fig. 11. From the slope and intercept values, the ΔH* and ΔS* values are 66.22 J mol^−1^ and −199.51 J K^−1^ mol^−1^, respectively.

## 4. Conclusion

Pure PbS and Cr-doped PbS films of optical homogeneity were deposited on the glass substrate *via* chemical bath deposition followed by annealing process for 2 h. The Cr incorporation decreases the average PbS crystallite size from 59.97 to 37.59 nm without changing its cubic structure or its preferential growth along (111) orientation. The absorbance is decreased and *E*_g_ values are increased with increasing Cr doping ratio. The photoelectrochemical behaviors of the Cr-doped PbS photoelectrode were studied under white and monochromatic light illumination in 0.3 M Na_2_SO_3_ electrolyte solution. The maximum ABPE value was 1.44% at 0.68 V and 390 nm. The optimum IPCE value was 13.5% at 390 nm. Also, the effect of temperature from 30 to 70 °C on the PEC behaviors was addressed and the thermodynamic parameters were estimated. The optimized electrode showed low activation energy *E*_a_ = 24.32 kJ mol^−1^, enthalpy ΔH* = 66.22 J mol^−1^, and ΔS* = −199.51 J K^−1^ mol^−1^, which interprets the efficient usage of the optimized electrode for PEC H_2_ generation.

## Conflicts of interest

No conflicts of interest.

## Supplementary Material
